# Case review of perinatal deaths at hospitals in Kigali, Rwanda: perinatal audit with application of a three-delays analysis

**DOI:** 10.1186/s12884-017-1269-9

**Published:** 2017-03-11

**Authors:** Aimable Musafili, Lars-Åke Persson, Cyprien Baribwira, Jessica Påfs, Patrick Adam Mulindwa, Birgitta Essén

**Affiliations:** 1 Paediatric and Child Health Department, University of Rwanda, Kigali, Rwanda; 2 Department of Women’s and Children’s Health, International Maternal and Child Health (IMCH), Uppsala University, Akademiska Sjukhuset, Uppsala, SE-751 85 Sweden; 3 Center for International Health, Education, and Biosecurity (CIHEB), Institute of Human Virology, University of Maryland, School of Medicine MGIC-Rwanda, KG, 6 AV no 22, Kigali, Rwanda; 4 Muhima District Hospital, Kigali, Rwanda

**Keywords:** Perinatal audit, Three-delays model, Urban hospitals, Rwanda

## Abstract

**Background:**

Perinatal audit and the three-delays model are increasingly being employed to analyse barriers to perinatal health, at both community and facility level. Using these approaches, our aim was to assess factors that could contribute to perinatal mortality and potentially avoidable deaths at Rwandan hospitals.

**Methods:**

Perinatal audits were carried out at two main urban hospitals, one at district level and the other at tertiary level, in Kigali, Rwanda, from July 2012 to May 2013. Stillbirths and early neonatal deaths occurring after 22 completed weeks of gestation or more, or weighing at least 500 g, were included in the study. Factors contributing to mortality and potentially avoidable deaths, considering the local resources and feasibility, were identified using a three-delays model.

**Results:**

Out of 8424 births, there were 269 perinatal deaths (106 macerated stillbirths, 63 fresh stillbirths, 100 early neonatal deaths) corresponding to a stillbirth rate of 20/1000 births and a perinatal mortality rate of 32/1000 births. In total, 250 perinatal deaths were available for audit. Factors contributing to mortality were ascertained for 79% of deaths. Delay in care-seeking was identified in 39% of deaths, delay in arriving at the health facility in 10%, and provision of suboptimal care at the health facility in 37%. Delay in seeking adequate care was commonly characterized by difficulties in recognising or reporting pregnancy-related danger signs. Lack of money was the major cause of delay in reaching a health facility. Delay in referrals, diagnosis and management of emergency obstetric cases were the most prominent contributors affecting the provision of appropriate and timely care by healthcare providers. Half of the perinatal deaths were judged to be potentially avoidable and 70% of these were fresh stillbirths and early neonatal deaths.

**Conclusions:**

Factors contributing to delays underlying perinatal mortality were identified in more than three-quarters of deaths. Half of the perinatal deaths were considered likely to be preventable and mainly related to modifiable maternal inadequate health-seeking behaviours and intrapartum suboptimal care. Strengthening the current roadmap strategy for accelerating the reduction of maternal and neonatal morbidity and mortality is needed for improved perinatal survival.

**Electronic supplementary material:**

The online version of this article (doi:10.1186/s12884-017-1269-9) contains supplementary material, which is available to authorized users.

## Background

Around 4.8 million perinatal deaths occur each year in the world, and, of these, 98% take place in low- and middle-income countries [1, 2]. Perinatal deaths consist of stillbirths or fetal deaths from 22 weeks of gestation and early neonatal deaths or deaths within the first week after birth [3]. Perinatal mortality is regarded as a key indicator, reflecting the quality of healthcare provided to women during pregnancy and childbirth as well as to neonates in the first week of life [4].

Perinatal audit and feedback is a clinical approach widely utilized to improve quality of care and to reduce perinatal mortality [5]. The audit process provides an opportunity to learn from critical events in the management of care of pregnant women and neonates. Healthcare workers are prompted to change their routines once they are given feedback about the inadequate practices that lead to these events [6]. Perinatal audit also explores factors occurring at home or in the community that were likely to contribute to the outcome. A meta-analysis of studies on perinatal audit conducted in low- and middle-income countries suggested that up to 30% of perinatal deaths could be averted after the implementation of a perinatal audit [7].

The three-delays model is considered to be a suitable framework for identifying and assessing the barriers faced by pregnant women before they access appropriate care. This approach was initially conceived to explore factors leading to maternal deaths. Three categories of factors are identified: delay in making decisions in seeking care (phase-one delay), delay in reaching the health facility (phase-two delay), and delay in receiving appropriate care at the health facility (phase-three delay) [8]. This model has also been applied to understand factors related to perinatal and neonatal mortality [9, 10].

In 2012, the rate of perinatal mortality at public health institutions in Rwanda was 29/1000 births [11]. According to the Demographic and Health Survey conducted in 2015, almost all women (99%) attended antenatal care. Among them, 44% attended four times or more. The proportion of women giving birth at health facilities was 91% [12].

In 2009, the maternal mortality audit process was implemented and routinely carried out at hospital level throughout Rwanda [13]. Two years later, the neonatal mortality audit process was also integrated into hospital practices [14]. However, a perinatal audit is not yet routinely performed. There is a need to also focus on factors related to fresh stillbirths as most of these are similar to the factors contributing to early neonatal deaths in this context [15]. Further, obstetric complications leading to maternal deaths are also common causes of perinatal deaths [16]. Thus, performing a perinatal audit may help to link knowledge between both the maternal and neonatal mortality audits.

The aim of this study was to carefully monitor births and outcomes (macerated or fresh stillbirths, live births, early neonatal deaths) at two Kigali hospitals, to identify factors causally related to perinatal deaths and to relate these to the three-delays factors experienced by delivering women; (1) mother’s inadequate healthcare-seeking behaviour, (2) difficulties in reaching a health facility, or (3) receipt of suboptimal medical and nursing care at the health facility, and to assess whether these deaths were potentially preventable.

## Methods

### Study design

The study adopted a perinatal audit design [4]. The audit was conducted prospectively and was related to the cases of perinatal deaths that had occurred among babies born in two urban hospitals.

### Study setting

This study was part of a programme that was aimed at analysing the causes and determinants of perinatal mortality at a district hospital (DH) and a tertiary referral hospital (TRH) from 18 July 2012 to 8 May 2013. These two hospitals, located in Kigali, Rwanda, assisted in almost 7900 and 2200 deliveries in 2012, respectively. The DH conducts the most deliveries in the country, whereas the TRH serves as the largest referral and University teaching hospital.

During the study period, the midwifery, nursing and medical teams working in the maternity and neonatal units included 79 midwives and nurses, 14 general practitioners, 3 obstetricians, and 2 paediatricians in the DH, whereas there were 78 midwives and nurses, 4 obstetricians, 2 paediatricians, 5 residents in paediatrics and 15 in obstetrics and gynaecology in the TRH. Most patients received in the two hospitals lived in the capital, which had approximately 1 200 000 inhabitants in 2012. Other patients came from the surrounding areas of the city or were referred from other urban or rural health facilities.

Patients attending both hospitals were mainly covered by the community health insurance system, as shown in our previous study [17]. Patients usually followed referral steps starting from public health centres or posts, then to district hospitals, and finally, to tertiary level hospitals. Those who bypassed these referral pathways were not covered by the community health insurance system, except in emergencies. Other existing health insurance systems cover civil servants, the military, and people working in the private sector. These insurance systems frequently covered patients who attended the TRH. Patients who had no insurance cover needed to pay all hospital charges themselves.

### Study population

Stillbirths and early neonatal deaths, which together constitute perinatal deaths, among births at the two hospitals were consecutively included in the study. A stillbirth was defined as a fetal death after at least 22 completed weeks of gestation or weighing 500 g or more at birth. An early neonatal death was defined as a live birth dying within the first seven days of life, the early neonatal period, and born after at least 22 completed weeks of gestation or weighing 500 g or more at birth [18]. Deaths that occurred among babies born outside the study sites were excluded from the study.

### Data collection

In each hospital, one nurse, one midwife, and one doctor were recruited and trained over two days on the study’s aim and data collection procedures, and how to conduct interviews with bereaved mothers and healthcare providers involved in the study using a questionnaire containing both closed and open-ended questions.

The first part of the questionnaire included information on the socioeconomic characteristics of the mothers and their households, such as household assets, maternal education and place of residence, and health insurance coverage, as well as the demographic characteristics of the mothers and their babies, such as parity, maternal and gestational age, birth weight, and sex. The second part included data on routine antenatal screening and care, pregnancy complications and their management, characteristics of labour and delivery, condition of the baby at birth (alive or stillbirth) and afterwards, Apgar score, management of the newborn’s illness that led to a fatal outcome, and time and causes of death.

A fetus that died during labour or delivery was defined as a fresh stillbirth. These stillbirths were also labelled intrapartum-related stillbirths as most of them were due to intrapartum-related insults or injuries [19]. When a death occurred before the onset of labour or delivery with signs of a degenerative process, the fetus was classified as a macerated stillbirth [18]. The time of fetal death in relation to admission was also recorded.

The third part of the questionnaire comprised information obtained from interviews with mothers and healthcare providers. Mothers were encouraged to describe the chain of events leading up to the death, including both clinical and social elements, such as reasons for seeking care at a health facility, the onset and development of symptoms, actions taken, barriers encountered when seeking care, and the clinical attention received at the health facility. Important dates and time-related events were recorded. When mothers were not available, information was sought from their partners or closest relatives. Healthcare providers also provided additional narratives about the circumstances that led to the death.

Data collected through interviews were systematically cross-checked against the information extracted from clinical records. When discrepancies were noted, interviews were repeated or other information sources were re-checked. Random repeat interviews were regularly performed to reinforce the quality of the data.

Narratives summarizing the information collated from the different sources were prepared for each case of death by the main investigator. The actual data collection was preceded by one month of piloting of the study procedures. The main investigator rigorously supervised the fieldwork, assisted by one doctor from each hospital.

### Audit meetings

Members of the audit committee were recruited from the hospitals and included two obstetricians, two paediatricians, and the main investigator, who is also a paediatrician. The main investigator introduced the study objectives and procedures at the first meeting. He also presented all cases of perinatal deaths that were discussed in each session, which was scheduled according to the availability of the members.

These presentations were based on the individual narratives and were followed by open discussions to identify the causes of the deaths, the underlying factors, and any potentially avoidable deaths. A single cause of death was assigned to each case. The avoidable factors were classified based on the three-delays model [8]. In some instances, several delays were identified for an individual case. The discussions were aimed at evaluating whether the delay had possibly or likely contributed to the fatal outcome. This process was consistent with previous evaluation studies completed elsewhere [4].

The quality of care provided to mothers and neonates was judged against evidence-based practices, as expressed in local guidelines and protocols or in the scientific literature [20]. The adequacy of care was also related to feasibility and the availability of resources and opportunities, such as any investigations in making a diagnosis, and the availability of drugs, consumables, equipment, and infrastructure.

A death was considered avoidable if improved management using available resources and opportunities would have altered the outcome. This approach has been applied in other reports on perinatal audit in low- and middle-income countries [21, 22]. In addition, the timing of death in relation to the mother’s admission to the hospital provided important orientation regarding the prevention of death. Audible fetal heartbeat on admission increased the likelihood that death would have been avoided.

The auditors discussed each case to reach a consensus. In case of disagreement, all sources of information were rechecked and conclusions were made during the subsequent meeting. The senior obstetrician had the final word in matters of pregnancy, labour, and delivery-related events, while the paediatrician made the final decision regarding neonatal care and mortality [23].

### Data analysis

The socio-demographic and clinical characteristics of the mothers and their babies included in the study were summarised in descriptive tables. Based on the individual narrative stories for each case of death, the auditors assessed the contributing factors that could be attributed to the three delays as well as to potentially avoidable deaths. Factors contributing to the same phase of delay were grouped together and listed in descriptive tables. Each phase of delay was illustrated by a case study that described barriers to access adequate maternal and neonatal care. Potentially avoidable deaths and their frequencies were described. IBM SPSS Statistics version 20 (IBM Corporation, Armonk, NY) was used for descriptive statistics.

The Rwanda National Ethics Committee granted ethical approval for this study (ethics approval no. 086 RNEC/2012, Kigali, Rwanda).

## Results

### Characteristics of participants

**Fig. 1:**
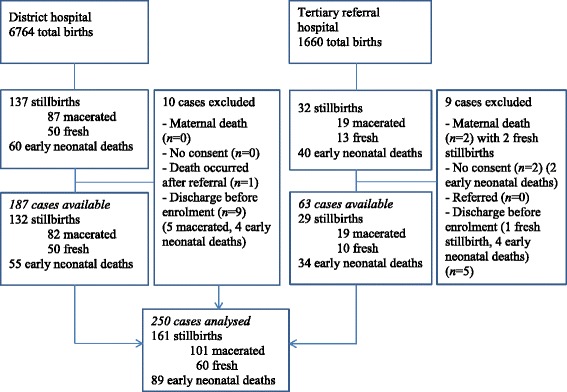
Flowchart of study population selection

Out of 8424 births, there were 269 perinatal deaths (106 macerated stillbirths, 63 fresh stillbirths, 100 early neonatal deaths) corresponding to a stillbirth rate of 20/1000 births and a perinatal mortality rate of 32/1000 births (Fig. 1). Singleton and twin births constituted 94 and 6% of the cases, respectively. Nineteen cases of deaths were not reviewed due to lack of information as the mothers had been discharged (*n* = 14) or died (*n* = 2) before interviews or refused to participate in the study (*n* = 2). One neonate died shortly after referral to another hospital and was also excluded from the study.

On admission, fetal heartbeats were inaudible for all macerated stillbirths and audible in 40% of the fresh stillbirths.

Table 1 shows the characteristics of mothers, pregnancies, deliveries, and children among the cases of perinatal death that occurred during the study period. Most babies were born to women living in urban areas and having a primary educational level. Most babies were born to women living with partners. The median maternal age was 28 years (range 16–47). Most women were multipara (parity 1–4). Most babies were born to women who had attended at least one antenatal care visit and one-fifth were born to mothers who had attended the recommended four antenatal care visits. Vaginal delivery was the most frequent mode of delivery. The highest proportions of deaths (53%) consisted of preterm births.

**Table 1:** Maternal, pregnancy, and childbirth characteristics for the cases of perinatal deaths at two hospitals in Kigali, Rwanda July 2012–May 2013

Characteristics	Perinatal deaths	
	*n*	%
Residence		
Urban	225	90
Rural	25	10
Maternal education		
Secondary or higher	91	36
Primary	142	57
No formal education	17	7
Marital status		
Living with partner	206	82
Living without partner	44	18
Maternal age at childbirth (years)		
< 20	14	6
20 − 34	182	73
> 34	54	21
Parity		
0	106	42
1 − 4	125	50
> 4	19	8
Gestational age (weeks)		
22 − 27	40	16
28 − 33	63	25
34 − 36	31	12
> 36	116	47
Antenatal visits		
0	15	6
1 − 3	188	75
> 3	47	19
Mode of delivery		
Spontaneous vaginal delivery	170	68
Caesarean section	76	30
Vacuum-assisted delivery	4	2

Maternal morbidity during pregnancy and labour is summarized in Table 2. Most babies, that is to say, 156/250 (62%), were born to mothers who had no complications during pregnancy. The most common medical problems identified in the remaining mothers were hypertensive disorders (preeclampsia, eclampsia, and pre-existing hypertension) and HIV/AIDS.

**Table 2:** Maternal morbidity during pregnancy and labour among the cases of perinatal death at two hospitals in Kigali, Rwanda July 2012– May 2013

Maternal diseases and complications	Perinatal deaths	
	*n*	%
Hypertensive disorders^a^	40	16
HIV/AIDS	30	12
Bleeding^b^	18	7
Chorioamniotitis/sepsis	7	3
Diabetes mellitus^c^	4	2
Uterine rupture	4	2
Malaria	2	1
Severe anaemia	1	0
Others^d^	3	1
All	94	38
None	156	62
Total	250	100

Note that the total of individual diseases/complications (*n* = 109) was higher than that of deaths (*n* = 94) because women could have several diseases/complications

^a^ Hypertensive disorders: preeclampsia (*n* = 32), pre-existing hypertension (*n* = 5) eclampsia (*n* = 3)

^b^ Bleeding: placenta abruption (*n* = 13), placenta praevia (*n* = 4)

^c^ Diabetes mellitus: gestational (*n* = 3) or pre-existing (*n* = 1)

^d^ Others: Asthma (*n* = 2), cardiac disease (*n* = 1)

The causes of perinatal mortality were categorized according to the type of death and phase of delay identified during the audit meetings (Table 3). For most cases of macerated as well as fresh stillbirths the cause of death was unknown. Among the suspected causes, asphyxia due to maternal hypertensive disorders (preeclampsia, eclampsia, and pre-existing hypertension) was the most common, followed by malformations for macerated stillbirths and umbilical cord prolapse or looping around the neck for fresh stillbirths. The major causes of early neonatal deaths were intrapartum-related neonatal causes, followed by prematurity or low birth weight and neonatal infections.

**Table 3:** Causes of perinatal mortality according to the type of perinatal death and phase of delay identified during audit meetings at two hospitals in Kigali, Rwanda July 2012– May 2013

Causes of deaths	Perinatal deaths		Cases with phase-one delay^a^	Cases with phase-two delay^b^	Cases with phase-three delay^c^
	*n*	%			
Total	250	100	98	24	93
Causes of macerated stillbirths					
Hypertensive disorders^d^	11	11	7	3	8
Malformations	6	6	0	0	0
Preterm pre-labour rupture of membranes	5	5	3	1	2
Placenta abruption	2	2	0	0	1
External trauma	1	1	1	0	0
Unknown	76	75	62	3	7
Total	101	100	73	7	18
Causes of fresh stillbirths					
Hypertensive disorders ^d^	11	18	5	2	9
Umbilical cord prolapse/loop around neck	10	17	3	1	4
Placenta abruption	7	12	1	1	3
Prolonged labour/obstructed labour	6	10	0	0	2
Uterine rupture	4	7	0	1	3
Malformations	3	5	0	0	0
Preterm pre-labour rupture of membranes	3	5	1	2	2
Unknown	16	26	3	1	10
Total	60	100	13	8	33
Causes of early neonatal deaths					
Intrapartum-related neonatal deaths	34	38	4	2	26
Prematurity/Low birth weight	30	34	5	5	12
Infections	11	12	3	2	2
Malformations	8	9	0	0	0
Neonatal jaundice	1	1	0	0	1
Unknown	5	6	0	0	1
Total	89	100	12	9	42

Note that each perinatal death could have had more than one type of delay

^a^ Phase-one delay: delay in decision-making and seeking adequate care

^b^ Phase-two delay: delay in reaching health facility

^c^ Phase-three delay: delay in receiving appropriate care at health facility

^d^ Hypertensive disorders: preeclampsia, eclampsia, pre-existing hypertension

Of the women whose pregnancies ended in macerated stillbirths, most had experienced a phase-one delay, whereas those who had fresh stillbirths or early neonatal deaths more frequently had a phase-three delay. A phase-two delay was less common in all groups of perinatal deaths.

### Factors contributing to delays

Potentially avoidable factors related to the three delays were identified in 79% (197/250) of perinatal deaths. Several factors could contribute to one case of death. Thus, the total number of factors contributing to mortality was higher than that of deaths (Table 4).

**Table 4:** Delays experienced by pregnant women and/or their babies in accessing appropriate care at health facility, as reported by the panellists during perinatal audit at two hospitals in Kigali, Rwanda July 2012–May 2013

Delays	Cases with delays
Phase-one: care-seeking	98
Lack of recognition of danger signs	81
Poor uptake of or compliance to formal care	25
Phase-two: reaching health facility	24
Lack of money	15
Long distance	6
Lack of health insurance	3
Social or family reasons	3
Phase-three: quality of care at health facility	93
Before admission	
No or late diagnosis	9
Inadequate management	
Delay in the referral^a^	13
Inadequate care or monitoring	12
After admission	
Late diagnosis	3
Inadequate management	
Inadequate monitoring or late intervention	41
Non-adherence to guidelines or best practices	5
After delivery	
Inadequate management	
Insufficient care	11
Inadequate monitoring	3
Total	197

^a^ The delays in referrals were observed when women were referred either from a health centre or private clinic to the district hospital or from a private clinic or the district hospital to the tertiary referral hospital. Note that there were more factors contributing to mortality than cases of deaths because several factors categorized as the same or different phases of delay could be identified in one case of death

#### Delay in seeking appropriate care: Phase-one delay

As shown in Table 4, phase-one delay (delay in decision-making to seek appropriate care) was more frequent than other phases of delay and was identified in 39% (98/250) of deaths. The most prominent reason for this delay was failure to recognize or report danger signs that occurred during pregnancy or labour. This delay was identified in 81 cases. The most frequently reported danger sign consisted of poor or absent fetal movements and signs of preterm labour such as preterm contractions or preterm rupture of membranes.

Some women were not aware of the severity of the danger signs and preferred to stay at home waiting for spontaneous resolution of these problems. Others had asked for advice from a partner, relative, neighbour or friend or had sought help from private pharmacies or traditional healers. The decision to seek care at health facilities had often been taken late, when danger signs had worsened or failed to subside.

Five women, who sought informal care providers, received traditional medicines because the main cause of danger signs was attributed to *ifumbi*. This was vaguely described as a clinical condition with abnormal symptoms varying from simple discomfort to major complications. The users of traditional medicines believed that *ifumbi* could be effectively cured by traditional medicine.

Four women had also used traditional medicines during labour, both before and after admission. The women considered these as effective stimuli of uterine contractions. Some also believed in the potential protection of these medicines against any witchcraft that could negatively influence maternal and fetal outcomes. Case study A (Additional file 1) illustrates a situation where the lack of knowledge on the severity of danger signs, combined with the utilization of traditional medicines, contributed to a phase-one delay.

Poor uptake of care by registered healthcare providers was noted in 16 cases. In two cases, the pregnancies were reportedly unintended and no antenatal care was sought. In two other cases, women were single mothers and did not have time to seek antenatal care due to a heavy workload to earn their living. Six women, including two with poor obstetrical histories (previous perinatal death), did not attend follow-up visits during pregnancy. Three of them explained that they could not return because they had not been clearly briefed by the healthcare provider about the need to attend. Others failed to give clear reasons for their non-attendance at follow-up visits. Five women initially wanted to deliver at home and came late to the health facility, when complications had already occurred. One woman refused the induction of labour suggested by her doctor despite having preterm rupture of the membranes and oligohydramnios.

#### Delay in reaching a health facility: Phase-two delay

The factors related to phase-two delay – when a woman decided to seek care but was delayed in reaching a health facility due to various reasons, such as lack of transport or limited revenue – were found in 10% (24/250) of cases (Table 4). Women reported lack of money as a key problem that delayed their timely care-seeking. They mainly expressed difficulties in mobilising money to cover the cost of transport or other expenses not covered by health insurance during their hospital stay. An example of delay that was ascribed to the lack of money is provided in case study B (Additional file 2).

Long travel distance (with a mean duration of 90 min) from home to the health facility or between health institutions was also reported in six cases of perinatal death. For four women, the long distance was compounded by a long walk or their having to travel by bike or motorbike on roads with poor conditions. For three women, the process of renewing or purchasing health insurance was undertaken late, which resulted in a delay to reach the health facility. Delayed access to care also occurred for two women because of illness in the family. One woman did not want to seek care during the night because it would not be convenient for her.

#### Delay in receiving appropriate care at a health facility: Phase-three delay

Various circumstances that led to the phase-three delays – in relation to suboptimal care received once at a health facility – were found in 37% (93/250) of deaths, as shown in Table 4.

Before admission, most cases of death were related to delays in women’s referrals from health centres or private clinics to district hospitals or from the district to the tertiary referral hospital. Such delays were identified in 12 cases, where death was subsequent to intrapartum-related hypoxia, preterm rupture of membranes, or preterm labour. One of these deaths was related to uterine rupture and occurred at a health centre after prolonged labour. In another case, a woman who had signs of preterm labour reportedly had a long waiting time (4 h) at a health centre before being examined and referred to the district hospital.

Late diagnosis and failure to diagnose or perform proper management were other common inadequacies in the practices of healthcare providers. In five cases of poor obstetric history (previous perinatal death), these women did not receive any special attention during pregnancy. In one case, gestational diabetes was diagnosed late in the pregnancy by providers at a district hospital in a woman who had initially been attending antenatal care clinics at a health centre. In another case, a woman referred from a health centre for poor progress of labour also had an umbilical cord prolapse, which was detected upon arrival at the hospital.

Similar delays in diagnosis or management had occurred for some women presenting with hypertension or preeclampsia. Another woman who also had gestational diabetes had received insufficient treatment at a private clinic. Substandard provision of care was observed in another woman at 37 weeks of gestation who was treated by a nurse at a health centre with an anti-inflammatory drug for one week due to multiple joint pain. Another case was related to umbilical cord prolapse that occurred at a health centre after an amniotomy was performed.

After admission, insufficient labour and fetal monitoring, and delay in taking appropriate action during the management of pregnancy or labour complications were the most frequent factors related to the phase-three delay. These inadequacies were found in 40 cases where the deaths were linked to various conditions, mainly prolonged or obstructed labour, preterm labour, maternal hypertension, preeclampsia and placenta abruption. These conditions also included two cases of uterine rupture that occurred in hospital. There were difficulties in handling one case of breech presentation and another case of vacuum-assisted delivery. In both cases, the doctor was called to assist after unsuccessful efforts by a midwife or nurse to perform the delivery. A caesarean section was delayed for 30 min because two other caesarean sections were ongoing and no other operating theatre was available in the hospital.

Non-adherence to the national guidelines was another barrier that delayed access to appropriate care in hospital after admission. An accidental double-dose of intravaginal misoprostol (100 mcg) was repeatedly administered to two women for labour induction. This could have led to the hyper-stimulation of uterus and fetal heart, causing or aggravating fetal hypoxia, which was the cause attributed to the death.

An attempt at vaginal delivery of a twin pregnancy was made in another woman, where ultimately a caesarean section was urgently performed due to the failure of delivery by vaginal route, which subsequently caused fetal asphyxia. An early caesarean section would have prevented this asphyxia, because the first twin had presented in a breech position. The national guidelines suggest a caesarean where the first twin has not presented in a cephalic position.

Late diagnosis of some pregnancy-related complications was another contributing factor to the phase-three delay, which was identified in two cases of placenta abruption and one case of preeclampsia. In these cases, the delay in making the diagnoses also affected the initiation of adequate treatment, as depicted in case study C (Additional file 3).

After delivery, insufficient newborn resuscitation and management of neonates with severe intrapartum-related hypoxia constituted major causes of suboptimal care. In some instances, resuscitation was not promptly initiated within the first minute after birth.

Some equipment, including suction tubes and facemasks for ventilation, were used inappropriately or were not in good condition when resuscitation began. In two cases, ventilation with bag and mask was applied in two neonates before proper skin drying and stimulation. Upon arrival to the neonatal intensive care unit, they were still wrapped in the wet cloths used when drying the skin at birth. In one of these cases, the endotracheal intubation was delayed due to the limited skills of the healthcare provider who was performing the advanced resuscitation.

The management of some cases of intrapartum-related hypoxia was temporarily hampered by a lack of second-line treatment such as phenytoin. Some of these cases also required the use of a ventilator machine, equipment that was not available in one of the two study hospitals. Two cases of death occurred while the transfer of the infant to a higher level of care was being discussed.

Another important suboptimal care factor consisted of the irregular monitoring of vital signs. This was observed in three preterm babies who developed hypothermia and bradycardia. In the three remaining cases, suboptimal care factors were related to inadequate management of some neonatal emergency conditions, including jaundice, feeding of a sick preterm baby, and delay in blood transfusion. The jaundice had eventually evolved into kernicterus. The inappropriate enteral feeding of one preterm baby presenting with food intolerance and frequent vomiting led to pulmonary aspiration.

### Avoidable deaths

Of 250 perinatal deaths reviewed, 51% were considered likely avoidable and 70% of them were fresh stillbirths and early neonatal deaths. When considering fresh stillbirths and early neonatal deaths alone, 60% of them were potentially preventable.

## Discussion

### Main findings

This perinatal audit revealed that a number of factors contributed to delays, which, in total, were identified in more than three-quarters of perinatal deaths. The most frequent delays were related to inadequate maternal care-seeking during pregnancy and childbirth (phase-one delay), followed by suboptimal care factors that occurred at health facilities (phase-three delay). Barriers encountered after deciding to go to a health facility (phase-two delay) were less common. Delays in seeking formal care were mainly due to the lack of recognition of danger signs. Delays in referrals, poor labour and fetal monitoring, and delayed action during the management of obstetric emergencies were the most predominant factors assessed at health facilities. Lack of money was the most common reason for not reaching the health facility in time. Approximately half of the perinatal deaths were judged to be potentially avoidable and most of these were fresh stillbirths and early neonatal deaths.

### Strengths and limitations

This perinatal audit study was the first of this kind carried out in Rwanda and included two main urban hospitals, which together manage a high number of complicated pregnancies. The risk of recall bias by mothers or healthcare providers was judged to be low, given that they were interviewed shortly after delivery or neonatal death, and before discharge from hospital.

The mothers included in this study predominantly lived in urban settings, mainly in the capital. Thus, the results may not be generalizable to the country as a whole due to varying social and health services conditions.

Making a distinction between miscarriage and stillbirths could be difficult, especially when the last menstrual period was unknown. We tried to minimize this bias by applying the standardized definition of 500 g as the lower limit of birth weight for stillbirths. Ultrasound findings were also used for dating pregnancies. Most women with suspected intrauterine fetal deaths underwent this examination to confirm death and to assess whether there was any visible malformation, and, in addition, gestational age and estimated weight were obtained at this time.

Another potential bias was the misclassification of fresh stillbirths as early neonatal deaths. However, this bias was minimal because each birth was attended by a skilled healthcare provider, who also assessed the signs of life after delivery [24].

No autopsy was performed to further investigate the cause of death. This implies that the number of deaths attributed to congenital malformations may in fact be higher. The classification of lethal congenital malformations were made by clinical examination, and all (*n* = 17) were considered non-avoidable [25].

Our investigation was, in principle, limited to data that were available at the two study hospitals. We were not able to access detailed information from other healthcare providers or facilities that had been involved in the management of the pregnant and birthing mothers. This is a limitation in that we were not able to obtain a full description of the quality of care provided to the women before they were admitted to the study hospitals.

### Comparison with other findings

The particularity of this study was the application of a perinatal audit with a three-delays model to explore factors underlying mortality in all perinatal deaths, regardless of age or weight at birth. Recently, a study carried out in one regional hospital in Tanzania employed the same approach but the study population was restricted to perinatal deaths weighing 2000 g or more [9]. Many other investigators in low-, middle- and high-income countries have limited the audit either to suboptimal care factors occurring at a health facility [26, 27] or to specific groups of perinatal deaths based on gestational age or birth weight [23, 28–30].

The factors related to the three phases of delay in accessing care were identified in 79% of all deaths in this study. This proportion is similar to that (80%) found by authors in Tanzania during a perinatal audit performed at a tertiary referral hospital [29]. These figures are higher than those reported from studies in 10 European countries, the Netherlands, and Uganda, where the factors contributing to mortality were assessed to be found in 46, 32 and 20% of deaths, respectively [4, 27, 30]. However, the latter three studies had applied different methodologies and definitions as compared to our study. The two studies conducted in the Netherlands and Uganda were also restricted to suboptimal care factors occurring at health institutions. In addition, the quality of care in each country or setting and the expert panel involved in the perinatal audit may also explain the different proportions of factors contributing to perinatal mortality. Evidence has shown that the levels of agreement on suboptimal care factors may vary between auditors [29, 31].

Mothers’ delay in decision-making to seek appropriate care was assessed as the most common contributing factors to perinatal mortality, followed by suboptimal care at hospital. However, the frequency of these delays was not consistent across the three groups of perinatal deaths (Table 3). Phase-one delay (care-seeking) was the most frequent in the group of macerated stillbirths, whereas phase-three delay (substandard care practices) was predominant among the groups of fresh stillbirths and early neonatal deaths.

An important reason to delay care-seeking was a lack of recognition of pregnancy or labour danger signs as well as social conditions, such as living without partner, unintended pregnancy, and misunderstandings between healthcare seeker and provider. These social stigma-related factors affecting the timing of presentation to skilled care before or during labour were also found in earlier studies carried out in low- and middle-income countries [22, 32, 33].

Barriers could be worsened by cultural beliefs, which might prompt women to seek care from traditional healers and misuse traditional medicines, as seen in our study. Studies conducted in Nigeria on stillbirth and in Uganda on uterine rupture showed that the intake of traditional medicines reportedly had a detrimental effect on maternal and fetal health [34, 35].

Our research indicated that phase-two delay (reaching health facility) was less common than other delays. The plausible explanation for this result was that most women included in the study (90%) lived in urban settings, where transport and health facilities were readily available. However, some issues, mainly lack of money and, less common, a long distance from home to health facility or between health facilities, lack of insurance and other social constraints, were linked to the phase-two delay. We have previously shown that lack of health insurance was associated with increased risk of perinatal deaths [17].

Studies in Uganda on neonatal mortality and in India on stillbirths and neonatal deaths showed that barriers to access the health facility were less common. These barriers included lack of transport, financial-related issues, lack of autonomy in decision-making in the absence of a partner, and a lack of guardians for children when mothers were not at home [10, 33]. In many other low- and middle-income countries, long distances, poor roads, lack of transport, lack of financial means, particularly in poor households, and lack of insurance have also been listed as key barriers to reach health facilities [36–39].

A recent systematic review on avoidable factors contributing to maternal and perinatal mortality in low-income settings showed that substandard practices by healthcare providers were the first contributing factors to death, followed by patient-related delays [22]. These findings are consistent with those reported in many other studies on perinatal audit [23, 29, 31]. A number of studies in these countries [9, 23, 40] have further highlighted the pivotal contribution of substandard care to intrapartum-related stillbirths and early neonatal deaths, which also was the case in our study.

Delay in referral from lower to higher levels of care [33], inadequate antenatal care, delay of diagnosis, poor management of high-risk pregnancy [7, 27, 34, 41], inadequate labour and fetal monitoring [9, 23, 29, 32], and insufficient newborn resuscitation and management of preterm births [15, 30] have frequently been reported as major contributors to suboptimal care by healthcare providers. These factors were also similar to those identified in our study.

The audit committee in this study concluded that almost half of the perinatal deaths reviewed could have been prevented. Several studies on perinatal audit conducted around the world have shown that the proportions of potentially avoidable deaths could vary between 14 and 75% [28, 29, 42, 43]. The differences in preventable deaths observed across these studies could be attributed to the levels of mortality or quality of care in each setting as well as the definitions and methods applied.

This study has shown that 60% of fresh stillbirths and early neonatal deaths could have been averted. This proportion falls in between those reported by internal (58%) and external (83%) auditors in a similar study conducted in Sudan [31]. In another audit conducted in Tanzania, the proportion of fresh stillbirths and early neonatal deaths considered avoidable was estimated at 64% by internal auditors versus 79% by external auditors [29]. However, in the latter study, the audit was performed on deaths weighing at least 1500 g.

### Policy implications

The proportion of potentially avoidable deaths was relatively high, particularly among fresh stillbirths and early neonatal deaths, suggesting that there is considerable room for improved care and perinatal survival. However, in order to better assess the potential for improvement regarding the quality of care and perinatal outcome, a continuous audit-feedback process would be recommendable in this setting.

This study has demonstrated that perinatal audit was feasible and potentially beneficial at hospital level in Rwanda, where previously it was non-existent. A large number of factors related to the three-delays model and underlying perinatal mortality was reviewed by an audit group formed by two obstetricians and two paediatricians. Thus, perinatal audit could even contribute to improved collaboration between providers of maternal and newborn care. Our analysis also showed that the perinatal audit could further strengthen the current national guidelines by linking this audit to the audits already in place for maternal, neonatal, and child mortality.

The need to reduce perinatal mortality calls for multilevel action to address the common delays affecting the timing of access to appropriate care during pregnancy and childbirth. This entails the strengthening of maternal education on the continuum of care across all stages of the life cycle [44]. Education on pregnancy and childbirth-related issues may increase awareness about danger signs and gradually break down the sociocultural barriers that may negatively influence health-seeking behaviour. Improved knowledge on reproductive health may also prevent women from relying on traditional healers and other unqualified providers as well as from the use of harmful practices such as the intake of traditional medicines [34, 35].

Special consideration should also be given to targeted interventions for vulnerable groups, including poor households and women living without partners, which, critically, may lack money or access to other assistance. Strategies encouraging the practice of making savings during the course of pregnancy and foreseeing difficulties that may hamper the access to care, such as the lack of insurance and transport fees, may also be of great interest for those involved in birth preparedness and other necessary care. of the proportion of women who attended at least one antenatal care visit in this population was low (94%) compared to the national average (99%) [12]. Thus, an in-depth investigation is needed to provide further understanding of the causes of non-compliance to the recommended antenatal care.

In 2004, Rwanda endorsed the road-map strategy for accelerating the reduction of maternal and neonatal morbidity and mortality [45]. This strategy is based on three pillars, including emergency obstetric and newborn care, delivery by skilled birth attendants, and family planning. The mechanisms for the operationalizing of this strategy need to be strengthened to ensure that these packages can be successfully scaled up. Particular attention should be given to early detection and proper management of high-risk pregnancies, compliance with referral policies to avoid unnecessary delays, improvement of labour and fetal monitoring, and timely decision for lifesaving interventions as well as birth preparedness. Incorporating the autopsy among the routine procedures and practices also proves to be essential for improving knowledge about the causes of perinatal deaths.

Additional efforts are required to improve the skills of staff involved in the management of the mother-newborn dyad through regular training and to ensure the availability of infrastructure and equipment. Strengthening of the supervision and motivation of staff needs to be addressed through the ongoing performance-based financing approach, among other strategies. Improving vital information on perinatal health and mortality may also serve as a basis for better planning for improved survival. Ultimately, to achieve positive and sustainable results, the collaboration within and between institutions in both public and private sectors should be emphasized.

## Conclusions

Perinatal audit combined with the three-delays model were applied to further understand contextual factors related to perinatal deaths occurring among delivering women in a low-income setting. The delays in care-seeking, followed by suboptimal care factors, were the most frequent barriers to accessing appropriate care. The delays in reaching health facilities were less common. A large number of potentially avoidable deaths were found among fresh stillbirths and early neonatal deaths and were related to modifiable factors. A multifaceted approach, including effective strategies such as the road map, for accelerating the reduction of maternal and newborn morbidity and mortality, especially targeting single mothers or those with no insurance, needs to be strengthened for better perinatal survival. This suggests the need for the improvement of intersectoral and multilevel collaboration among healthcare providers and other stakeholders.

## Additional files

Additional file 1: Case study A. (DOC 22 kb)
Additional file 2: Case study B. (DOC 22 kb)
Additional file 3: Case study C. (DOC 23 kb)

## Abbreviations

AIDS: Acquired Immune Deficiency Syndrome
DH: District hospital
HIV: Human immunodeficiency virus
TRH: Tertiary referral hospital
