# Supplementary material for: Case review of perinatal deaths at hospitals in Kigali, Rwanda: perinatal audit with application of a three-delays analysis
Source: BMC Pregnancy Childbirth. 2017 Mar 11;17:85. doi: 10.1186/s12884-017-1269-9 (PMC5346214; doi:10.1186/s12884-017-1269-9)
Supplement: Additional file 1: — Case study A. (DOC 22 kb) [file 12884_2017_1269_MOESM1_ESM.doc]

**Additional files**

Additional file 1 **Case study A**

A 26-year-old woman in her second pregnancy was referred from a health centre to hospital because of prolonged rupture of the membranes, abdominal pain, fever, and accelerated fetal heart rate (182 beats per minute). Her first pregnancy had ended in an intrapartum death at 39 weeks of pregnancy after a prolonged labour. During the current pregnancy, the woman had attended one antenatal care visit at a health centre. The rupture of the membranes had occurred at home five days earlier. At that time she sought care from a traditional healer, who provided indigenous medicines. The vaginal discharge was persistent and later became purulent with a foul smell. She developed abdominal pain and fever, which worsened over time. The uterine contractions started on the fifth day after rupture of the membranes. She decided to seek care at a health centre and was immediately referred to hospital A. On arrival, a doctor examined her, performed an ultrasound assessment and requested some laboratory tests. Chorioamniotitis was suspected and a caesarean section was urgently performed due to fetal distress. After taking samples for laboratory assessment, she was started on antibiotics. A term baby boy having 1- and 5-minute Apgar scores of 4 and 6, respectively, and weighing 2800 g was referred to the neonatal intensive unit after extensive resuscitation efforts. After three days, the baby died as a result of septic shock.
