# Supplementary material for: Case review of perinatal deaths at hospitals in Kigali, Rwanda: perinatal audit with application of a three-delays analysis
Source: BMC Pregnancy Childbirth. 2017 Mar 11;17:85. doi: 10.1186/s12884-017-1269-9 (PMC5346214; doi:10.1186/s12884-017-1269-9)
Supplement: Additional file 2: — Case study B. (DOC 22 kb) [file 12884_2017_1269_MOESM2_ESM.doc]

Additional file 2 **Case study B**

A 41-year-old woman in her sixth pregnancy, mother to four children, came directly from home to hospital on a motorbike, approximately 10 hours after the uterine contractions started. She decided to seek care at the hospital shortly after the rupture of the membranes. She could not leave home earlier because she was waiting for her husband, who had gone to borrow some money from a friend. She had a history of one abortion during her second pregnancy. She had gone for two antenatal care visits during the current pregnancy. The clinical examination performed on arrival revealed a non-pulsating umbilical cord prolapse. The ultrasound confirmed the absence of fetal heartbeats. The woman delivered a preterm baby boy that was a fresh stillbirth weighing 1500 g and having a gestational age estimated at 33 weeks.
