# Supplementary material for: Case review of perinatal deaths at hospitals in Kigali, Rwanda: perinatal audit with application of a three-delays analysis
Source: BMC Pregnancy Childbirth. 2017 Mar 11;17:85. doi: 10.1186/s12884-017-1269-9 (PMC5346214; doi:10.1186/s12884-017-1269-9)
Supplement: Additional file 3: — Case study C. (DOC 23 kb) [file 12884_2017_1269_MOESM3_ESM.doc]

Additional file 3 **Case study C**

A 19-year-old woman in her first pregnancy of 35 weeks was referred from a health centre to hospital for onset of preterm labour and hypertension. The uterine contractions had started at home and she arrived at the health centre three hours later. She was referred to the hospital after examination by a nurse. At hospital A, a clinical examination was done and a diagnosis of preterm labour was made. The woman was admitted to the labour ward for further monitoring and laboratory investigations. However, no blood pressure measurement was documented on admission. After 8 hours, oxytocin infusion was administrated to increase contractions. Four hours later, a midwife urgently called the doctor after noticing deceleration of the fetal heartbeats. The midwife was further alerted by high blood pressure that was mentioned on the referral sheet from the health centre. However, this referral sheet was not scrutinized until then. The doctor performed a reassessment of the woman. Her blood pressure measurement was 160/110 mmHg and severe preeclampsia was confirmed after a urine dipstick test that showed protein in her urine. Oxytocin infusion was stopped, intravenous hydralazine and oral nifedipine were administrated, and an emergency caesarean section was performed. A preterm baby boy weighing 2100 g was born with 1- and 5-minute Apgar scores of 3 and 4, respectively. Resuscitation with bag-and-mask ventilation was initiated at birth. Intubation was performed and oxygen and adrenaline were also administered, but the baby died 25 minutes later.
